# An implementation science study to enhance cardiovascular disease prevention in Mukono and Buikwe districts in Uganda: a stepped-wedge design

**DOI:** 10.1186/s12913-019-4095-0

**Published:** 2019-04-25

**Authors:** Geofrey Musinguzi, Rhoda K. Wanyenze, Rawlance Ndejjo, Isaac Ssinabulya, Harm van Marwijk, Isaac Ddumba, Hilde Bastiaens, Fred Nuwaha

**Affiliations:** 1 Department of Disease Control and Environmental Health, School of Public Health, College of Health Sciences, Makerere University, Kampala, Uganda; 2 Department of Primary and Interdisciplinary Care, Faculty of Medicine and Health Sciences, University of Antwerp, Antwerp, Belgium; 3 Department of Medicine, College of Health Sciences, Makerere University, Kampala, Uganda; 4 Department of Primary and Interdisciplinary Care, Briton and Sussex University Medical School, Sussex, UK; 5 Department of Health, Mukono, District, Uganda

**Keywords:** Implementation, Risk prevention, Cardiovascular diseases, Uganda

## Abstract

**Background:**

Uganda is experiencing a shift in major causes of death with cases of stroke, heart attack, and heart failure reportedly on the rise. In a study in Mukono and Buikwe in Uganda, more than one in four adults were reportedly hypertensive. Moreover, very few (36.5%) reported to have ever had a blood pressure measurement. The rising burden of CVD is compounded by a lack of integrated primary health care for early detection and treatment of people with increased risk. Many people have less access to effective and equitable health care services which respond to their needs. Capacity gaps in human resources, equipment, and drug supply, and laboratory capabilities are evident. Prevention of risk factors for CVD and provision of effective and affordable treatment to those who require it prevent disability and death and improve quality of life. The aim of this study is to improve health profiles for people with intermediate and high risk factors for CVD at the community and health facility levels. The implementation process and effectiveness of interventions will be evaluated.

**Methods:**

The overall study is a type 2-hybrid stepped-wedge (SW) design. The design employs mixed methods evaluations with incremental execution and adaptation. Sequential crossover take place from control to intervention until all are exposed. The study will take place in Mukono and Buikwe districts in Uganda, home to more than 1,000,000 people at the community and primary healthcare facility levels. The study evaluation will be guided by; 1) RE-AIM an evaluation framework and 2) the CFIR a determinant framework. The primary outcomes are implementation – acceptability, adoption, appropriateness, feasibility, fidelity, implementation cost, coverage, and sustainability.

**Discussion:**

The study is envisioned to provide important insight into barriers and facilitators of scaling up CVD prevention in a low income context.

This project is registered at the ISRCTN Registry with number ISRCTN15848572.

The trial was first registered on 03/01/2019.

## Background

### Cardiovascular disease and risk factors on the rise

Globally, approximately one third of all deaths are attributed to cardiovascular disease (CVD) [1]. In Europe, CVD are estimated to be responsible for half of all deaths causing more deaths than any other condition [2]. Approximately, three quarters of all estimated global deaths due to CVD take place in low and middle income countries (LMICs) [3]. Moreover, it is projected that by the year 2030, CVD alone will be responsible for more deaths than infectious diseases (including HIV/AIDS, tuberculosis, and malaria), maternal and perinatal conditions, and nutritional disorders combined in LMICs [4]. The high and increasing burden of CVD in developing countries is attributable to urbanization and higher levels of risk factors, low age of manifestation, large population sizes and the high proportion of young adults [3]. The eight modifiable risk factors including: history of hypertension or diabetes, obesity, unhealthy diet, lack of physical activity, excessive alcohol consumption, raised blood lipids and psychosocial factors are equally on the rise especially in LMICs [5]. The main CVD causing most death are heart failure, stroke, coronary heart disease as well as renal complications.

Sub-Saharan Africa (SSA) is experiencing a rapid epidemiological transition [3] and Uganda in particular has seen major causes of death shift from exclusively infectious diseases to a combination of communicable and non-communicable diseases (NCDs) [6]. The first nationwide NCD risk factor survey reported a prevalence of hypertension of 26.4%; 28.9% in urban and 25.8% in rural areas, among whom only 7.7% were aware of their high blood pressure status [7]. Authors allude that low awareness mirror a high burden of undiagnosed and un-controlled high blood pressure in the population [7]. In a study in central region focusing on Mukono and Buikwe districts, more than one in four adults were reportedly hypertensive [8]. Moreover, very few (36.5%) reported to have ever had a blood pressure measurement [8]. In the same districts, a trend analysis of health facility data revealed increasing burden of CVD at these facilities with cases of ischemic, rheumatic and chronic heart diseases reportedly on the rise. Likewise, stroke, hypertension and diabetes mellitus are on the rise [9].

### Preparedness of the healthcare system

The burden of CVD in LMICs is compounded by a lack of integrated primary health care programs for early detection and treatment of people with increased risk. As a result many people in LMICs who suffer from CVD have less access to effective and equitable health care services which respond to their needs [10]. Consequently, many individuals are detected late in the course of the disease and die younger from CVD, often in their most productive years. At the household level, emerging evidence suggest that CVD are contributing to poverty due to catastrophic health spending and high out-of-pocket expenditure [10]. At the macro-economic level, significant proportions of national health budgets for LMICs are directed towards managing CVD. Moreover, managing these conditions is a daunting task in LMICs [10]. High burden of disease, socioeconomic barriers and inequalities in access to treatment, suboptimal staffing including shortage of physicians at health-care facilities and limited capacity to conduct investigations are widely documented as limiting factors [11–15].

In Uganda, the healthcare system is oriented to manage communicable diseases and this has not changed significantly even with the changing trends in NCDs [16]. A 2013 NCD needs assessment found that none of the health facilities in Uganda met the World Health Organization (WHO) standard for essential tools and medicines needed to implement effective NCD interventions [17]. Moreover, capacity gaps in human resources, equipment, drug supply, and laboratory capabilities were evident [17].

In Mukono and Buikwe districts, an assessment of the health system capacity to care and manage hypertension cases revealed a multitude of gaps both from the health provider and patient perspectives. The health workers highlighted that they had inadequate skills to manage hypertension and its complications, lacked relevant guidelines and equipment, were understaffed and the health facilities they worked in frequently suffered stock outs of antihypertensive [18]. The health workers also reportedly noted that high costs, adherence challenges, and transport problems affected patients uptake of hypertensive services [18].

### Interventions, strategies and models

Combination prevention of risk factors for CVD and provision of effective and affordable treatment to those who require it prevent disability and death and improve quality of life. In the current study, we are drawing lessons from the HIV response in combination with the Innovative Care for Chronic Conditions (ICCC) framework to implement contextually appropriate interventions that address prevention of CVD in Mukono and Buikwe districts in Uganda. The ICCC framework is an adaptation of the chronic care model (CCM) but has mainly been tested in high income countries [19]. The framework expands the policy environment and puts more emphasis on the role of the community, to enable its applicability in a low income context [20]. A combination of both HIV/AIDS response models which enjoy extensive application in SSA and the ICCC framework provide synergies for a comprehensive study on CVD prevention in a low income setting.

### Evidence for the proposed interventions, strategies and models

Innovative HIV/AIDS response strategies have prevented 8 million deaths and 30 million new infections since 2000, and many of the worst-affected countries have managed to cease—and in some cases, reverse—the spread of the disease [21]. In SSA, a steady reduction in HIV incidence and AIDS-related deaths is clearly documented [22]. Although the fight against HIV/AIDS is not over and remains an important priority, the HIV/AIDS response model provides unprecedented evidence that chronic care in LMICs is feasible amidst scarce resources. Successful HIV models [23] responsible for the celebrated milestones in SSA have been tested in several countries including Tanzania, South Africa, Zambia, Uganda, and Malawi [22]. As reported by Olmen and colleagues, the innovative responses are an interplay of three pillars: a public health approach (PHA); community and peer support; and strengthening the health services in which care is embedded [22]. The main principles of the PHA are simplification of treatment protocols and clinical monitoring; decentralization of ART care delivery to the local health centre and community level; and task shifting and involvement of community and people living with HIV (PLWHA) in program design, management, and care [22]. In the current project, we will borrow from these models to implement a comprehensive package for prevention of CVD. At the community, we train community health workers (locally referred to as Village Health Teams (VHTs) to conduct health education, risk assessments, and motivational interviewing; facilitate creation of or support strengthening of community networks; and promote cardiovascular health and education. At the primary healthcare level, we shall identify and train non-physicians (mainly clinical officers/nursing staff) to screen for risk factors and provide basic care and management of CVD risk factors; train peers counsellors to support patient follow-up, conduct peer counselling and risk assessment; and provide basic buffer supplies to ensure continuity of CVD prevention and control services at the level. We integrate a mobile and e-health component to improve data capture, storage and patient follow-up.

## Study objectives and research questions

### General objective

The general objective of the study is to implement an enhanced CVD prevention program and evaluate the implementation process and determine the effectiveness on improving profiles for people with intermediate and high risk factors for CVD at the community and health facility levels.

### Research questions

- How effective is an enhanced community approach in improving population knowledge and screening for CVD risk factors, referral and enhancing lifestyle change in a real world setting versus usual care? What is the uptake and what factors influence implementation?
- How effective is an enhanced primary healthcare approach in improving CVD risk profiles of patients attending healthcare facilities? What is the uptake in routine practice and what factors influence implementation?
- What is the utility of an e-health screening tool in profiling for CVD risk factors at the community and at primary healthcare level? What is the uptake and what factors influence implementation uptake?
- What is the impact of an enhanced comprehensive CVD prevention program on patient outcomes versus usual care?

## Methods/design

### Study design

**Fig. 1:**
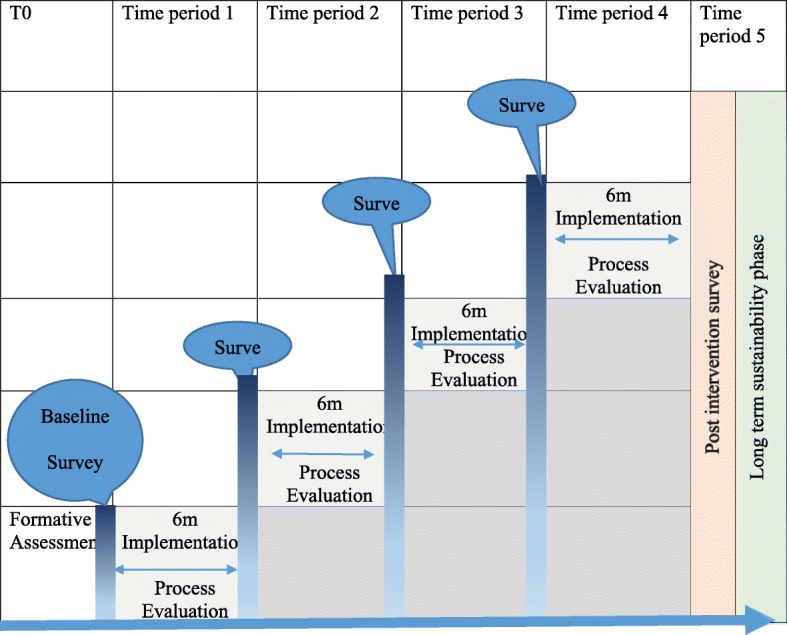
Illustration of a stepped-wedge study design with periodic surveys every 6 months

The overall study is a type 2-hybrid stepped-wedge (SW) design that emphasizes both clinical effectiveness and implementation outcomes, with the eventual goal of more rapid translation and uptake to usual care settings. The design employs mixed methods with iterative improvement cycles [24]; and data collection entailing repeated measures at the community, household, health facility and patient levels. The proposed design involve incremental execution and adaptation and allows continued measurement of key process and outcome indicators [24]. Sequential crossover take place from control to intervention until all clusters are exposed (Fig. 1). The core analyses will be qualitative and we will feed quantitative data into these to further our understanding.

### Context, study sites and sequencing

**Fig. 2:**
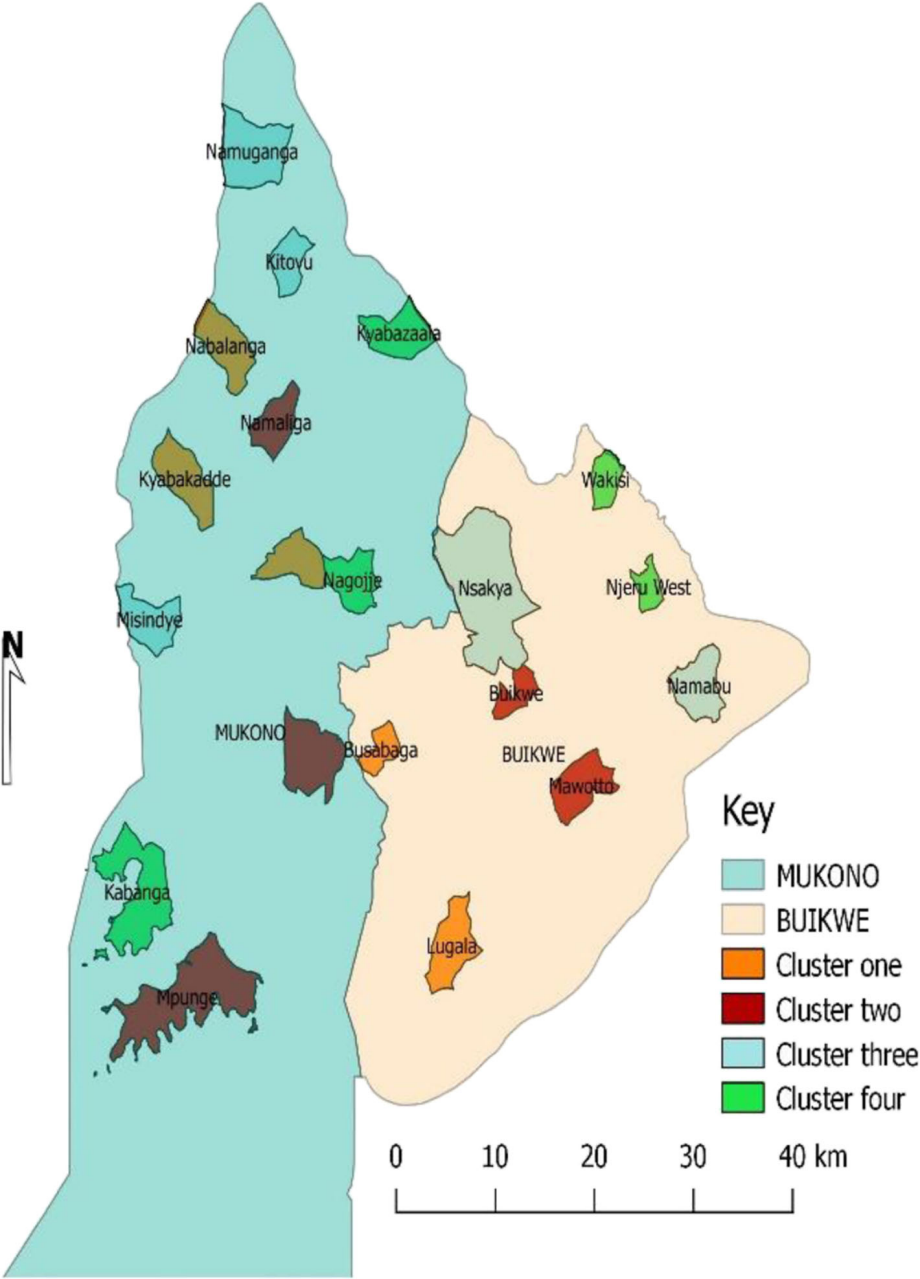
Map of Mukono and Buikwe showing selected parishes for the stepped wedge study

The study will take place in Mukono and Buikwe districts, home to more than 1,000,000 people. The two districts are largely rural with a significant proportion (25%) living in urban or semi-urban neighbourhoods. The districts share common boarders and lie along the main transport corridor connecting Kampala (the capital city) through Nairobi to Mombasa Sea Port in Kenya. The districts are strategically located between the two biggest urban areas of Jinja and Kampala. The prevalence of hypertension is high in these districts and only 35.6% of the adult general population have ever had their blood pressure measured. More than one in four of the general adult population are hypertensive [25] and yet the health system is very weak to adequately care for them [26]. This context provides an opportunity to scale-up proven, safe, efficient and cost-effective interventions to mitigate CVD risk factors. Mukono and Buikwe districts together have 7 health sub districts, and 23 sub-counties (15 in Mukono and 8 in Buikwe). Regarding health facilities, the two districts have more than 126 health facilities of which 40.5% are government owned, 41.3% are private for profit (PFP) and the rest are private not for profit (PNFPs). Of the facilities, 5 are hospitals (1 public), 4 health Centre IVs (3 public) and 23 are HCIIIs (20 public). For this study, 20 parishes (Fig. 2) hosting the 20 public health centre IIIs in Mukono and Buikwe will be included. From each Parish, the HCIII is enrolled for the Health facility study and four randomly selected villages are enrolled for the community study. In the stepped wedge, five parishes are initially assigned to the intervention (cycle 1) and the rest in usual standard of care (control). Every 6 months, additional five parishes (health facilities and villages) are switched to intervention till all are exposed at the end of the 4 cycles. We include Kawolo Hospital in Buikwe and Kojja and Mukono HCIVs in Mukono to provide a referral mechanism for complicated cases. Minimal support including training is provided to these levels by the project.

### Description of formative activities, and intervention plans

From October 2017, the study team conducted a situational analysis of the CVD burden and contextual factors in Uganda with a focus on Mukono and Buikwe districts. The study revealed gaps (reported earlier) and a range of contextual recommendations to improve CVD prevention. The recommendations from the analysis are summarized as follows: empower lower level health facilities to manage CVD and risk factors, utilize volunteers in screening for CVD using non-laboratory means, utilize expert clients in health promotion activities and peer support, institutionalize peer model for care and management and improve data capture for CVD and create database.

Drawing from the recommendations and in the context of global literature on best practices (reported earlier), we propose to advance CVD prevention along the cascade through health promotion and education and screening for risk factors at the community (community plan) and strengthen health services at the primary healthcare level (Primary healthcare plan) in Mukono and Buikwe district in Uganda. This study is part of a bigger project code named “SPICES”. SPICES is an acronym for Scaling up Packages of Interventions for Cardiovascular disease prevention in selected sites in Europe and sub-Saharan Africa. The partners implementing SPICES and their respective countries are: University of Antwerp, Belgium (Coordinator); Brighton and Sussex Medical School and Nottingham Trent University, both in the United Kingdom; Brest University in France, and the University of Limpopo in South Africa.

### Primary healthcare plan

Facilities in the intervention cycle(s) are strengthened with certain aspects to improve screening, diagnosis and management of risk factors for CVD. These aspects include: supplies and equipment such as blood pressure (BP) monitoring devices, glucometers and buffer stock for first line anti-hypertensive medicines; guidelines and standardized treatment protocols; computers and digital devices to improve data capture and storage; and training health workers and patients peers. Health workers are trained based on a manual adopted with modification from the Uganda Ministry of Health training manuals and others materials on Non communicable diseases. Trained health workers are equipped with a simplified algorithm (adopted with modification from the hearts and the WHO PEN protocols and the Uganda Clinical guidelines) to guide them in screening, diagnosis and managing the risk factors. Meanwhile, peers are equipped with knowledge and skills to conduct peer counselling, support screening activities and follow-up of patients.

### Community plan

The community plan revolves around working with community health workers (VHTs), existing networks and community structures to promote knowledge, improved lifestyles, risk assessment and cardiovascular health. CHWs are trained to conduct health education, promote lifestyle change through motivational interviewing and goal setting techniques, screen for risk factors at the community using non laboratory tools, and conduct home to home visits. Existing community networks and community structures are identified and supported to promote improved lifestyle behaviours, screening for risk factors and cardiovascular health education and promotion. Electronic and print media (messages) are circulated to enhance health promotion and improved lifestyle behaviours.

### Data capture and information management

To enhance data capture and information management and storage, both the community and health facility plans are supported with an e-health and mobile health platforms. The electronic plan will be explored to understand its contribution in improving data management and screening for CVD risk factors, enhancing linkage and referral as well as triggering behaviour change. Electronic enhancement entail mounting data capture and screening tools on a mobile/computer platform and used for registration, risk assessment and where possible linking/confirming linkage of participants for service.

### Evaluation plan

To evaluate the study, we propose to conduct a series of cross-sectional surveys every 6 months at time (T) 0, T6, T12, T18 and T24 at the community and health facility levels. We assess with key community and household members, health facilities, health workers, and patients. The evaluations uses both: qualitative assessments – to evaluate the process, barriers and opportunities; and quantitative measurements – to assess effects and process outcomes. During the interventions, formative processes (process evaluations) and detailed documentation of process, costs and activities take place.

### Inclusion and exclusion

#### Inclusion

- Both male and female
- Aged 18 years and above
- Consent to participate in the study

#### Exclusion

- The mentally ill will not be included in the evaluations
- Non consenting adults

### Sample size and sampling

#### Community quantitative surveys

For the community series of cross sectional surveys, we adopt the formula by Hemming et al. 2016 for cross sectional sample size determination for a stepped wedge study. To determine a moderate effect size of 0.3 (SD 1) for say change in Blood pressure at 5% significance, and detectable power of 88%, before and after the exposure to the intervention, given a sample size that is feasible and realistic in these setting, with four (4) steps and five (5) clusters per step, a total sample size of 50 participants per cluster is assumed reasonable [27]. However, assuming an intracluster correlation coefficient, r, of 0.01, a fixed cluster sample size, m = 50, survey rounds, t = 5 and a design effect of 2.93 [27] with 20 predetermined clusters; the required total sample size per survey round is 2930 households. We recognise that a random sample of household in a vast geographic setting can return a lot of non-responses. Thus, to cater for non-responding households and to avoid replacement of randomly selected eligible households, we factor in a non-response of 30% and generate the final household sample of 4000; with each cluster contributing 200 households. *Sampling households and participants*: The sampling frame for the surveys is generated through a mapping and listing exercise. All households in the catchment area are assigned a unique number to generate a sampling frame. A random sample of 200 eligible households with at least one adult per household is generated from the sampling frame using a random number generator. The selection is conducted by the study statistician. Study personnel (trained research assistants) approach the selected households for recruitment into the household/community survey. When a selected household is approached and has at least one eligible adult, study personnel describe the purpose of the study in the appropriate language, obtain participant consent and administer the study questionnaire and conduct anthropometric and blood pressure measurements using standardised and validated equipment. All eligible adults in the household aged 25–70 years are enrolled.

#### Health facility surveys

All Health centre IIIs are assessed for readiness and capacity to provide care for CVD prevention and control. The assessment is conducted with a quantitative questionnaire adapted with modification from the World Health Organisation service availability and readiness assessment (SARA) instrument. The tool is administered to the facility/unit heads to capture the relevant information on CVD prevention, service availability and readiness and staffing. In addition, health workers’ knowledge, practices and attitudes are assessed to generate relevant skill and knowledge gaps during pre and post training assessments using a health workers questionnaire. All health workers enrolled for training participate in these evaluations. Furthermore, a purposive sample of health workers participate in qualitative evaluations to assess organizational readiness for change, including perceived barriers to change, acceptability, feasibility, and appropriateness of implementing the enhanced program at the health facility. The required sample size is informed by the principal of data saturation in qualitative research. For readiness of the community component, CHWs are assessed with a questionnaire to document their current knowledge, practices and attitudes on prevention of CVDs. In addition, perceived barriers to change, acceptability, feasibility, and appropriateness of implementation is explored qualitatively to garner CHWs perspectives. We also plan to conduct focus group discussions with community members and key informant interviews to assess community attitudes, perceptions, acceptability and appropriateness of community interventions and strategies. Similarly, an appropriate number of FGDs is determined based on principle of information saturation, geographical diversity, age distribution and gender and other parameters deemed relevant. ***Patient surveys*** – To generate information on the health status of the patients, quality of life, satisfaction, challenges, adherence practices and support mechanisms, proportion of screened patients at health facilities, a survey of patients is conducted using a patient assessment questionnaire.

## Study outcomes and evaluation framework

### Primary outcome

Implementation (Reach, Appropriateness, Acceptability, Self-efficacy, Adoption, Cost, Feasibility, Fidelity and Sustainability).

### Secondary outcome

Effectiveness (Change in selected profiles e.g. knowledge, blood pressures, waist hip ratio/BMI, alcohol history, smoking status, quality of life, etc.).

#### Evaluation frameworks

We propose to evaluate the project guided by two validated and widely used implementation frameworks 1) RE-AIM, an evaluation framework and 2) the Consolidated Framework for Implementation Research (CFIR), a determinant framework [28–32]. The broader implementation outcomes as proposed by Proctor and colleagues [33] —acceptability, adoption, appropriateness, feasibility, fidelity, implementation cost, coverage, and sustainability are pre-determined in order to understand the factors affecting the implementation, the processes, and the accruing results. The RE-AIM and CFIR frameworks are selected based on a review of literature that demonstrate that they are suitable for a complex intervention. The RE-AIM (Reach, Effectiveness, Adoption, Implementation and Maintenance) framework offers a comprehensive structure designed to systematically assess the robustness of interventions across settings and individual subgroups and the potential for their scaling up and spread to additional settings [30]. Reach, the percent and representativeness of individuals willing to participate; Effectiveness, the impact of the intervention on targeted outcomes and quality of life; Adoption, the per cent and representativeness of settings and intervention staff that agree to deliver a program; Implementation, the consistency and skill with which various program elements are delivered by various staff; and Maintenance, the extent to which individual participants maintain behaviour change long term and, at the setting level, and the degree to which the program is sustained over time within the organizations delivering it (www.re-aim.org). The CFIR is an implementation framework that allows for systematic evaluation of barriers and facilitators that may impact the adoption, implementation, and/or maintenance of a program [34–36]. It provides a useful template for organizing key concepts in implementation research. A detailed description of the framework is available elsewhere [34, 36]. Some elements of this framework will be used to guide data collection (both qualitative and quantitative), and analysis of the contextual factors that have potential to impact the implementation of the project. The CFIR Research Team provides resources to guide implementation evaluation (https://cfirguide.org/). In Table 1 we present a detailed process evaluation guide checklist that we have adopted with modifications of the frames to measure the delivery of the intervention and the evaluation process. Pre-implementation, implementation and post implementation processes of the intervention are foreseen using mixed methods [28].

**Table 1:** Evaluation framework for both the Community/Health facility Strategy

Primary outcomes - (Reach, Appropriateness, Acceptability, Self-efficacy, Adoption, Cost, Feasibility, Fidelity and Sustainability)			
Implementation outcome	Description	Measurement	Time points
Reach	Proportion of the health care/community providers/target population approached; Uptake of intervention packages; and proportion of adherent	Analysis of routine data generated by CHW, peers & Health facilities and individual/patients quantitative assessments using household/patient questionnaires	0, 6, 12, 18 and 24 months
Appropriateness	The extent to which proposed interventions can be delivered at health facilities/community	Individual interviews Focus group discussion, Key informant interviews	0, 6, 12, 18 and 24 months
	Organizational Readiness	Availability and functionality of infrastructure including personnel, equipment, supplies etc. measured using the Health facility readiness and capacity assessment questionnaire	0, 6, 12, 18 and 24 months
	Linkage to healthcare	1. Self-report using h/h questionnaire, 2. Data extraction checklist	12 and 24 months
	Referrals for task sharing/shifting	Data extraction checklist	12 and 24 months
Acceptability	User and provider feedback	Focus group discussions guides Key informants	Formative process ongoing
	Individual/Patient satisfaction (Needs)	Patient Satisfaction Questionnaire	12 and 24 months
Self-efficacy	Personnel beliefs about own competencies to achieve implementation goals	Provider questionnaire Pre/post training assessment tools	12 and 24 months
Adoption	Implementation of the project and challenges to implementation (Barriers and opportunities and coping mechanisms).	Focus group discussions and individual interviews Key informant interviews	0, 6, 12, 18 and 24 months (formative process ongoing)
Cost	Costs associated with implementing the packages	Checklists for cost data (Health facility cost data related to the project Community program cost data)	12 and 24 months
Feasibility	Exposure to and retention of the enhanced interventions e.g. CVD education, counseling etc.	Data extraction checklist (Daily activities conducted related to the enhanced interventions e.g. counselling, no. of people profiled, followed up etc.)	12 and 24 months
Fidelity	The extent to which providers are delivering packages as per the protocol/guidelines	Observer rating forms	Formative process ongoing
Sustainability	The extent to which the program is being implemented as a standard of practice	Key informant interviews with providers.	12 and 24 months
Secondary outcomes – Effectiveness (Change in selected profiles e.g. knowledge, blood pressures (diastolic blood pressure), waist hip ratio/BMI, alcohol history, smoking status, etc.) measured at T 0,6,12,18 and 24 month using surveys.			

### Data management and quality control

The study coordinator will be responsible for all data collection instruments and their transportation to and from the study sites in Mukono and Buikwe and back to Makerere University School of Public Health (MakSPH). The study instruments are either paper based or electronic collected with android enabled phones and tablets using REDCap. All data (paper based or electronic) before submission to MakSPH is field edited for accuracy and consistency. Clean electronic data will be synchronized by the field team and uploaded to the MakSPH server on a daily basis. All qualitative data will be audio recorded and transcribed verbatim and then securely stored at MakSPH. Qualitative audio transcript shall also be edited, coded and analysed at MakSPH. Completed paper based forms shall be converted to electronic through data entry at MakSPH. To ensure quality of data collected, the coordinator is supported by two field supervisors/data quality controllers to provide day to day support and supervision of the data collection exercise. The supervisors check progress and quality of work, clarify questions on study instruments, and supply additional study instruments and stationery, and advice on how to solve logistical problems. Further quality assurance and fidelity checks are conducted by the study investigators on a monthly or more regular basis.

### Data analysis

Qualitative data will be analysed using the framework analysis technique, which facilitates thematic analysis.

The quantitative analyses will be guided by the principle of intention-to-treat and clusters analysed according to their randomised crossover time irrespective of whether crossover was achieved at the desired time. The analysis will be conducted in Stata or R Software. Initially, descriptive analysis (before and after) will be conducted and categorical data presented using counts and percentages, while continuous variables presented using measures of central tendencies. No multiple imputations will be conducted for missing data. The following descriptive are envisioned.

1. Reach of the project, captured through: the number and percentage of providers and individuals enrolled into the project at the community and health facility levels; the number of individuals partaking and adhering to the recommended interventions at the various levels.
2. Effectiveness: Activities and clinical endpoints will be used to assess effectiveness of the project at selected intervals observed longitudinally. The measures include: the proportion of project activities (community/health facilities) achieved at any point in time; change in clinical profiles e.g. blood pressures, BMI, alcohol history, smoking status, quality of life etc., achieved at 0, 6, 12, 18 and 24 months; and proportion of completers and attritors.
3. Adoption: The number/percentages of individuals/providers approached or agreed to take part in the intervention (s) and proportion of individual providers (CHW, Peers or NPHW) that deliver the intervention.
4. **I**mplementation: Captured by detailed description of the contextual adaptations of the project, the time required for intervention, and the costs of the intervention. Implementation information including process, facilitators and barriers, costs, and contextual issues will be analysed descriptively and qualitatively. Readiness of implementation (Available resources) – BP monitoring devices, Weighing scales, Stadiometers, measuring tapes, Glucometers and strips, first line antihypertensive drugs, guidelines, personnel, space, time etc. will be descriptively described and presented using graphs. Quality and quantity of equipment and their functionality, supplies and other necessary infrastructure will be descriptively analysed. Fidelity – Evidence of use of guidelines and protocols/simplified algorithm and any iterations recorded will be analysed descriptively and qualitatively. Costs - Costs associated with implementing the packages. Monetary requirements for each intervention (community and health facility based) will be analysed descriptively as a measure of sustainability based on feedback reports from community members, patients, CHWs, health unit staff and project and field coordinators.
5. Maintain: The extent to which the project becomes part of routine practice and policy will be explored. Evaluation of maintenance will include the following: Analysing the proportion of health providers who are introduced to the enhanced approach and those adapting the strategies.

Paired t-tests will be run to compare means across clusters and within clusters. However, given the time trend and clustering effects, we proceed to adjust for different observation periods and for clustering in the data by fitting an appropriate generalised linear mixed model or using generalised estimating equations. For continuous (and normally distributed) outcomes, we fit a linear model with random effect for cluster and fixed effect for each step; and, for binary outcomes, a logistic regression model with random effect for cluster and fixed effect for each step.

## Discussion

The study is envisioned to provide important insight into effectiveness and barriers and facilitators of uptake of CVD prevention at the community and health facility levels in a low income context. The project will utilize both the Green Open Access and Gold Open Access model to disseminate and publish research data. Dissemination workshops and meetings will be organized at the local, national and international platforms. We will use available institutional repository to archive anonymised data. The project is the beginning of a long intervention programme which aim to translate the research findings including tested models into routine implementation.

## Trial status

The trial has received both approvals from the Makerere University Higher Degrees Research and Ethics and the Uganda National Council of Science and Technology. Permission to conduct the trial has also been obtained from both districts – Mukono and Buikwe. Currently, a baseline survey is ongoing and intervention start in the first quarter of 2019. The trial is registered with ISRCTN registry, number ISRCTN15848572.

## Abbreviations

CFIR: Consolidated Framework for Implementation Research
CHWs: Community Health Workers
CVD: Cardiovascular Diseases
ICCC: Innovative Care for Chronic Conditions
LMICs: Low and middle income countries
NCDs: Non-communicable diseases
PLWHA: People living with HIV
RE-AIM: Reach Effectiveness Adoption Implementation Maintained
SPICES: Scaling up Packages of Interventions for Cardiovascular disease prevention in selected sites in Europe and sub-Saharan Africa
SSA: Sub-Saharan Africa
VHTs: Village Health Teams
WHO: World Health Organisation
